# Congratulatory

**Published:** 1887-10

**Authors:** 


### CONGRATULATORY.

Dr. W. D. Miller has successfully passed his examination (the so-called "rigorosum") for the medical degree of the German Empire, and emerged with the predicate "Magna cum Lauda," his record being fourteen out of a possible fifteen. The latter number is almost never attained, the highest mark of any other candidate this year being eight. Dr. Miller not only has the congratulations of his associates of The Independent Practitioner, but his many other American friends will be glad to know of his distinguished success.

It is but natural that the graduates of American dental colleges should be looked upon with suspicion in Germany, and had so representative a man failed, it would have been a matter of reproach to and reflected discredit upon American dentistry. For this reason Dr. Miller has put forth every energy that he might pass with credit, and the work has absorbed the time that he gladly would have devoted to other studies. This journal has been deprived of the benefit of his writings; but now that the trying ordeal is over, Dr. Miller proposes again to take up his line of special investigations, and our readers will have the first chance to reap the advantages to be derived therefrom.

